# Electrochemical Synthesis of Polypyrrole/Cu_2−*x*_Se Composites for Enhanced Thermoelectric Performance

**DOI:** 10.3390/ma19163496

**Published:** 2026-08-18

**Authors:** Yunfei Cai, Caiyan Gao, Cun-Yue Guo

**Affiliations:** 1 School of Chemical Sciences, University of Chinese Academy of Sciences, Beijing 101408, China; caiyunfei24@mails.ucas.ac.cn; 2 Beijing National Laboratory for Molecular Sciences (BNLMS), Key Laboratory of Green Printing, Institute of Chemistry, Chinese Academy of Sciences, Beijing 100190, China

**Keywords:** polypyrrole, copper selenide compounds, electrochemical synthesis, flexible thermoelectric films, energy harvesting

## Abstract

Copper selenide compounds, owing to their excellent electrical transport properties and low thermal conductivity, are promising thermoelectric materials, but their poor mechanical flexibility limits practical applications in flexible devices. Herein, a simple all-electrochemical strategy was developed to fabricate polypyrrole (PPy)/Cu_2−*x*_Se composite thermoelectric films through sequential electropolymerization of pyrrole and electrodeposition of Cu_2−*x*_Se. By optimizing the deposition potential of Cu_2−*x*_Se and pyrrole polymerization time, the thermoelectric performance of the composite films was significantly enhanced. The optimized PPy/Cu_2−*x*_Se composite film achieved a maximum power factor of 174.05 ± 9.87 μW m^−1^ K^−2^, nearly 300 times higher than that of pristine PPy, while maintaining a low thermal conductivity of 0.30 W m^−1^ K^−1^. The composite film also exhibited excellent flexibility, retaining 94.77% of its initial power factor after 1000 bending cycles. A flexible thermoelectric device assembled from the composite films delivered a maximum output power of 240.5 nW at Δ*T* = 50 K. This work provides an effective strategy for developing Cu_2−*x*_Se-based flexible thermoelectric composites.

## 1. Introduction

Recent advances in artificial intelligence, the Internet of Things, and wearable electronics have accelerated the widespread adoption of distributed electronic devices in daily life [1,2,3,4]. Providing these miniaturized devices with a continuous, reliable, and maintenance-free power supply has therefore become a critical challenge limiting their further development. Harvesting ambient energy sources, such as solar, mechanical, and thermal energy, is considered one of the most promising approaches for constructing next-generation self-powered electronic systems [5,6,7]. Among these technologies, thermoelectric technology, which can directly convert a temperature gradient into electrical energy without requiring external power sources or moving components, has demonstrated great potential in wearable electronics, electronic skin, wireless sensor networks, and health-monitoring devices [8,9,10]. In particular, flexible thermoelectric devices are capable of continuously harvesting low-grade thermal energy from the small temperature difference naturally existing between the human body and the surrounding environment, thereby attracting increasing research interest [11]. Compared with conventional bulk inorganic thermoelectric materials, flexible thermoelectric materials are required not only to exhibit high thermoelectric performance but also to possess lightweight, excellent flexibility, and scalability for large-area fabrication. Therefore, developing flexible thermoelectric materials that simultaneously combine superior thermoelectric performance and mechanical flexibility has become an important research direction in the thermoelectric field [12,13]. The performance of thermoelectric materials is commonly evaluated by the dimensionless figure of merit *ZT* $\left(ZT=\frac{S^{2}\sigma T}{\kappa }\right)$, where *S* is the Seebeck coefficient, *σ* is electrical conductivity, *κ* is thermal conductivity, and *T* is absolute temperature [14,15,16,17].

Conducting polymers, such as poly(3,4-ethylenedioxythiophene) (PEDOT) and polyaniline (PANI), have emerged as important candidates for flexible thermoelectric materials owing to their intrinsically low *κ*, excellent mechanical flexibility, and good processability [18,19,20,21,22]. Among various conducting polymers, polypyrrole (PPy) has attracted considerable attention due to its relatively high *σ*, good environmental stability, low cost, biocompatibility, and facile controllable synthesis via electrochemical polymerization [23,24,25,26,27,28]. However, its relatively poor thermoelectric performance has limited its application in thermoelectric fields, motivating the development of effective strategies to enhance its thermoelectric properties [29]. Li et al. optimized the doping level of PPy through a two-step electrochemical post-treatment strategy and achieved the highest thermoelectric performance reported for pristine PPy at that time; nevertheless, the corresponding PF remained as low as 5.23 μW m^−1^ K^−2^ [30].

To further enhance the thermoelectric performance of PPy, increasing attention has been devoted to organic/inorganic composite strategies, in which PPy is combined with inorganic thermoelectric materials possessing superior electrical transport properties and high *S*. Through the synergistic effects between the two components, the overall thermoelectric performance can be effectively optimized. In recent years, significant progress has been achieved in PPy-based composites incorporating inorganic thermoelectric materials such as Te, Bi_2_Te_3_, and Ag_2_Se [31,32,33]. These studies indicate that the incorporation of inorganic thermoelectric components can not only enhance the *σ* and *S* of the composites, but also preserve the intrinsic advantages of conducting polymers, including excellent flexibility, lightweight characteristics, and facile processability. Therefore, constructing organic/inorganic heterostructures through the synergistic integration of conducting polymers with various inorganic thermoelectric materials has become an important strategy for developing high-performance flexible thermoelectric materials [34,35,36]. Nevertheless, current studies are still mainly focused on Te-based, Bi-based, and Ag-based thermoelectric systems, while the exploration of other inorganic thermoelectric materials with outstanding thermoelectric performance and cost advantages remains relatively limited.

Cu_2_Se, as a representative copper-based selenide thermoelectric material, has attracted extensive attention due to its excellent electrical transport properties, intrinsically low *κ*, and abundant elemental reserves [37,38,39]. Compared with conventional thermoelectric materials such as Bi_2_Te_3_ and Ag_2_Se, Cu_2_Se possesses advantages including lower raw-material cost and higher elemental abundance [40,41]. In addition, Cu_2_Se can be controllably synthesized at relatively low temperatures through electrochemical deposition, while PPy can also be grown in situ via electrochemical polymerization. This inherent compatibility in fabrication processes provides a promising route for constructing all-electrochemically fabricated organic/inorganic composite thermoelectric films. However, to the best of our knowledge, studies on PPy/Cu_2_Se composite thermoelectric materials have rarely been reported, and their microstructural characteristics, interfacial interactions, and thermoelectric properties still remain largely unexplored. However, copper selenide materials generally possess intrinsic defect chemistry, and Cu vacancies are frequently observed, resulting in non-stoichiometric compositions such as Cu_2−*x*_Se.

Based on the above considerations, PPy/Cu_2−*x*_Se composite thermoelectric films were fabricated in this work through a successive electrochemical deposition strategy. A conductive PPy layer was first prepared via galvanostatic electropolymerization, followed by the in-situ growth of a Cu_2−*x*_Se layer on the PPy surface through potentiostatic electrodeposition, thereby constructing an organic/inorganic composite structure with intimate interfacial contact. The effects of electrodeposition conditions on the composition, microstructure, thermoelectric performance, and mechanical stability of the composite films were systematically investigated. The optimized PPy/Cu_2−*x*_Se composite film achieved a PF of 174.05 μW m^−1^ K^−2^, a low *κ* of 0.30 W m^−1^ K^−1^, and an estimated *ZT* value of 0.17 at room temperature, while retaining 94.77% of its initial performance after 1000 bending cycles. In addition, the thermoelectric device assembled from the composite films exhibited stable power output at room temperature, demonstrating its promising potential for flexible thermoelectric energy-harvesting applications. A comparison with previously reported flexible thermoelectric films is provided in Table S1 of the Supporting Information to better illustrate the significance of this work. This work not only expands the compositional design space of PPy-based organic/inorganic composite thermoelectric materials, but also provides a simple and effective strategy for the electrochemical fabrication of flexible thermoelectric films.

## 2. Experimental

### 2.1. Materials

Unless otherwise stated, all commercially available reagents and solvents were used directly without further purification. Pyrrole (Py, 99%) was purchased from Shanghai Aladdin Biochemical Technology Co., Ltd. Selenium oxide (SeO_2_, 99%) and *p*-toluenesulfonic acid (*p*-TSA, 98%) were purchased from Beijing Innochem Science & Technology Co., Ltd., Beijing, China. Copper sulfate pentahydrate (CuSO_4_·5H_2_O, 99%) was purchased from Shanghai Macklin Biochemical Co., Ltd., Shanghai, China.

### 2.2. Characterization

The electrosynthesis and linear sweep voltammetry (LSV) measurements were conducted using an electrochemical workstation (CHI660E, Chenhua Instruments Co., Shanghai, China). Stainless steel (SS) was used as the working electrode for the electrochemical polymerization of pyrrole, followed by the electrodeposition of Cu_2−*x*_Se. Raman spectra were obtained using a Labram Odyssey Raman system (HORIBA Ltd., Kyoto, Japan) at an excitation wavelength of 532 nm. X-ray photoelectron spectroscopy (XPS) measurements were conducted with an ESCALAB QXi X-ray photoelectron spectrometer (Thermo Fisher Scientific Ltd., Waltham, MA, USA). X-ray diffraction (XRD) was conducted using a D8-ADVANCE diffractometer (Bruker, Ltd., Karlsruhe, Germany). Scanning electron microscopy (SEM) and energy-dispersive spectroscopy (EDS) analyses were performed using an SU8010 scanning electron microscope (HITACHI Ltd., Tokyo, Japan). High-resolution transmission electron microscopy (HRTEM) was performed using a field emission transmission electron microscope (HT-7700Exalens, Tokyo, Japan). TG measurements were carried out on a thermogravimetric analysis analyzer (TGA, STA200, HITACHI Ltd., Tokyo, Japan) over a temperature range from room temperature to 600 °C at a heating rate of 10 °C min^−1^. The thermal stability of the films was evaluated by TGA/DTA using approximately 5−10 mg of sample in an alumina crucible under a nitrogen atmosphere with a gas flow rate of 100 mL min^−1^.

The thermoelectric parameters of the films were tested at room temperature using MRS-3RT thermoelectric parameter test system (JouleYacht Ltd., Wuhan, China). The detailed measurement procedure is illustrated in Figure S1. The thickness of the composite thin film was measured by SEM. The error bars were calculated based on the standard deviation of at least three independent measurements to ensure statistical reliability. The out-of-plane thermal conductivity (*κ*_⊥_) was measured by a transient hot wire method using a thermal conductivity tester (TC3000E, Xiatech Electronics Co., Ltd., Hangzhou, China). The open-circuit voltage (*V*_oc_), short-circuit current (*I*_sc_), and output power of the thermoelectric generator (TEG) were tested at different temperature gradients using a VC890C multimeter (Yisheng Victory Technology Co. Ltd., Shenzhen, China). Unless otherwise specified, all structural and chemical characterizations, including SEM, TEM, XRD, Raman, XPS, and TGA/DTG measurements, were performed using the as-deposited films directly grown on SS substrates. The films used for thermoelectric performance measurements and flexible TEG assembly/testing were transferred onto adhesive tape to enable flexible device fabrication and evaluation.

## 3. Results and Discussion

### 3.1. Film Preparations

**Figure 1:**
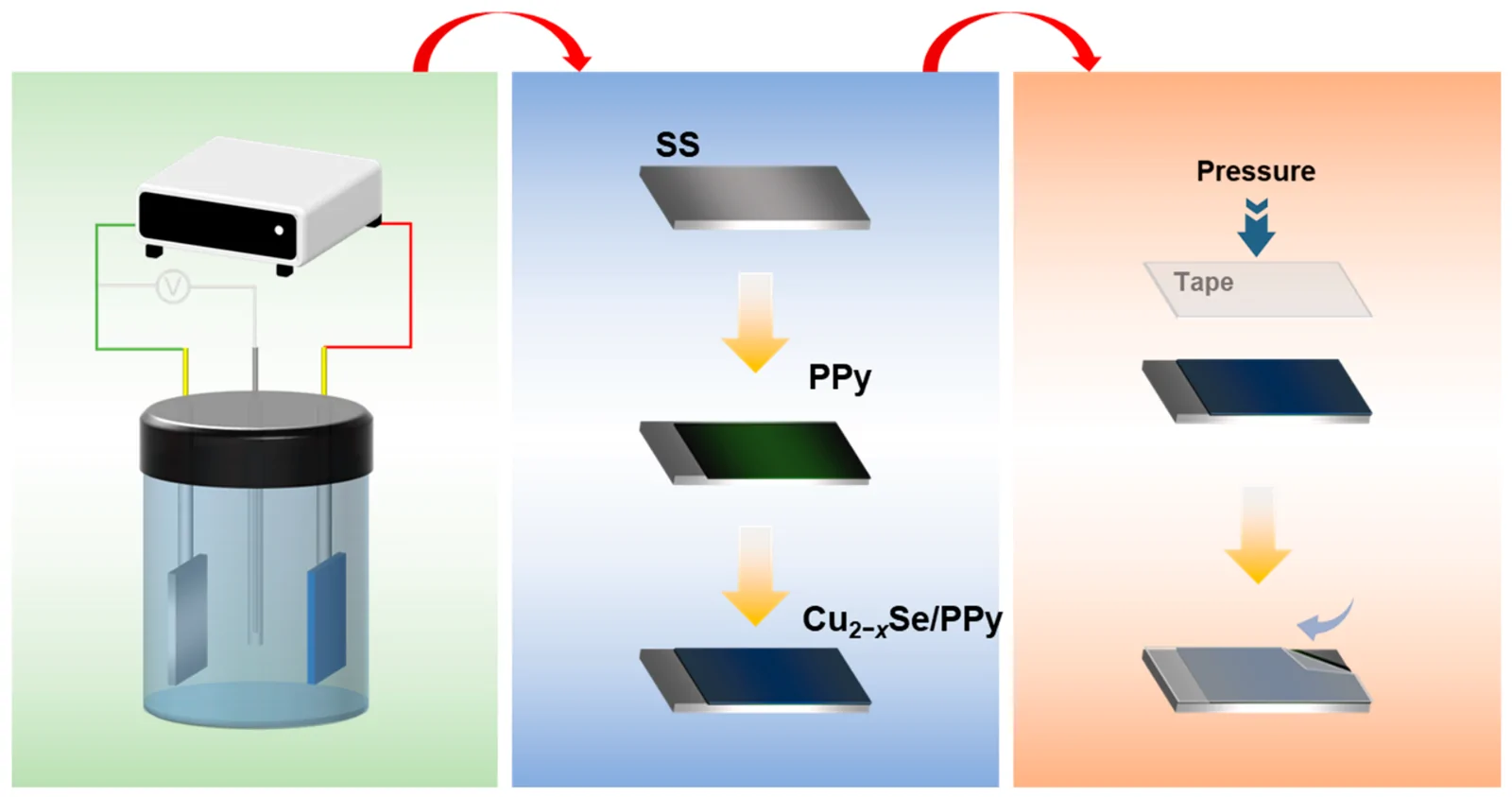
Schematic illustration of the electrochemical assembly of the PPy/Cu_2−*x*_Se composite film [42].

The electrochemical assembly of PPy/Cu_2−*x*_Se composite films involved two sequential processes, including the electropolymerization of pyrrole and the electrodeposition of Cu_2−*x*_Se. All electrochemical processes were carried out in a conventional three-electrode system similar to that reported in previous work [42], where an Ag/AgCl electrode was used as the reference electrode (RE), a platinum sheet (99.99%, 2 × 2 cm^2^) served as the counter electrode (CE), and the working electrode (WE) was adjusted according to the specific electrochemical reaction. It should be noted that the active area of all working electrodes was fixed at 4 cm^2^. The detailed fabrication procedure is schematically illustrated in Figure 1, while the specific experimental details are provided in the Supporting Information.

### 3.2. Electrochemical Behaviors

**Figure 2:**
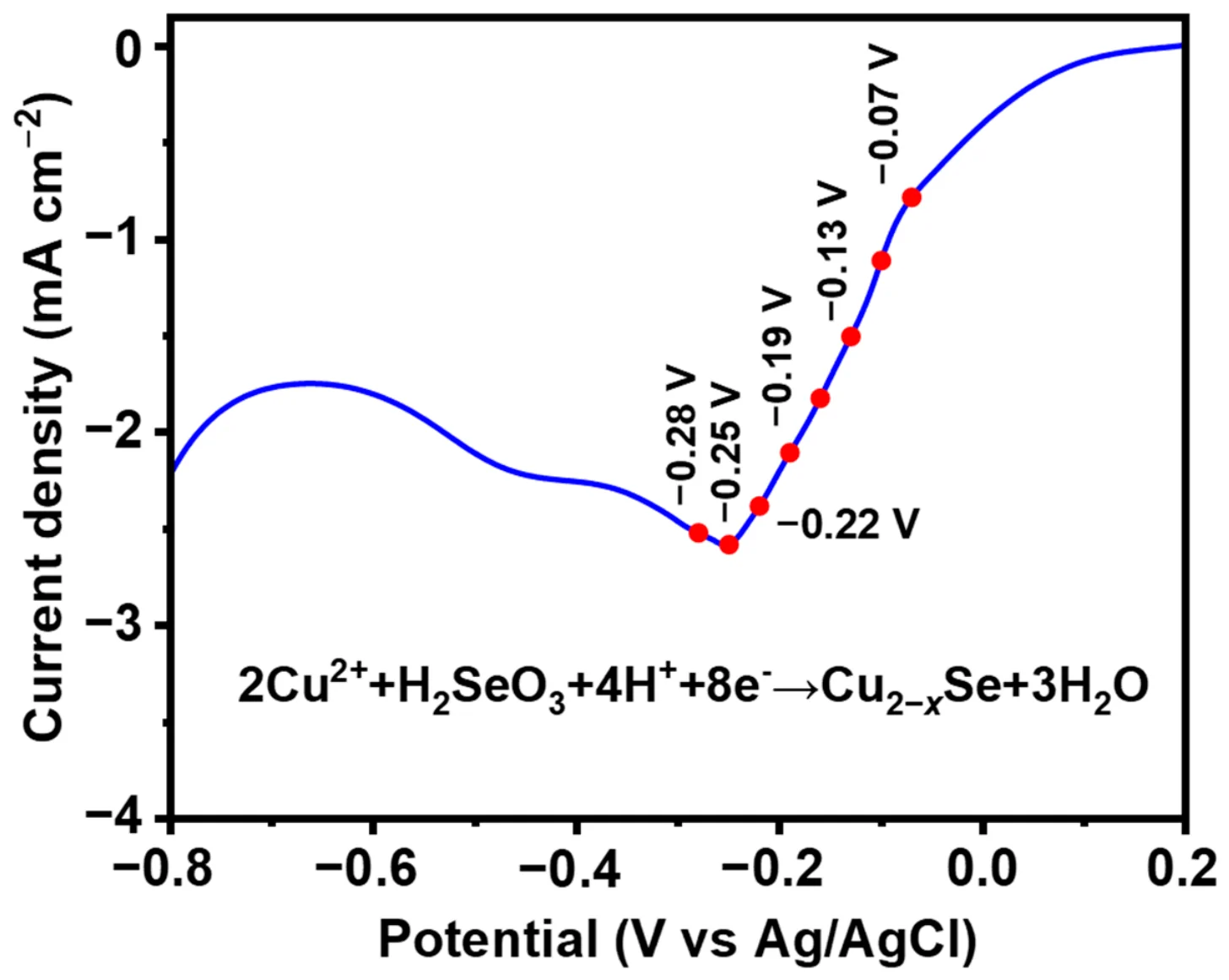
LSV curve of Cu_2−*x*_Se at a scan rate of 20 mV s^−1^.

To investigate the electrodeposition behavior of Cu_2−*x*_Se, linear sweep voltammetry (LSV) measurements were performed in an electrolyte containing CuSO_4_·5H_2_O and SeO_2_, and the results are shown in Figure 2. As the scanning potential shifted toward the negative direction, the cathodic current gradually increased. When the potential reached approximately −0.07 V, a noticeable increase in current was observed, indicating the onset of the Cu_2−*x*_Se electrodeposition process. The overall reaction can be described as follows [43,44]:

(1) $${2Cu}^{2+}+H_{2}SeO_{3}+{8e}^{-}+4H^{+}\to {Cu}_{2-x}Se+3H_{2}O$$

As the potential further shifted toward more negative values, the cathodic current increased rapidly, indicating an accelerated deposition rate of Cu_2−*x*_Se. Based on the LSV results, the deposition potential range for Cu_2−*x*_Se was selected from −0.07 to −0.28 V to further investigate the influence of deposition potential on the structure and thermoelectric performance of the PPy/Cu_2−*x*_Se composite films.

### 3.3. Morphology Characterization

**Figure 3:**
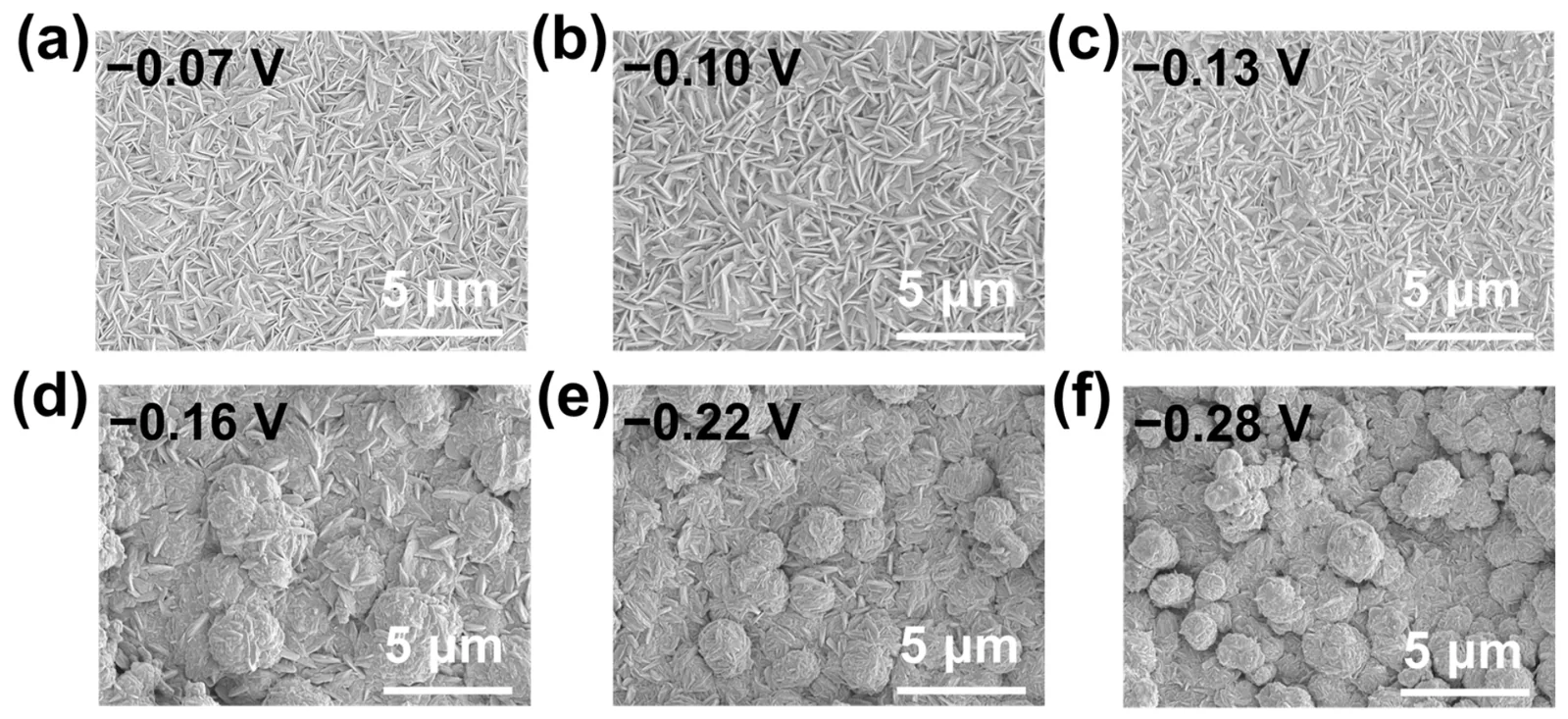
SEM images of PPy/Cu_2−*x*_Se composite films deposited at (**a**) −0.07 V, (**b**) −0.10 V, (**c**) −0.13 V, (**d**) −0.16 V, (**e**) −0.22 V, and (**f**) −0.28 V.

Figure 3 presents the surface morphologies of the PPy/Cu_2−*x*_Se composite films prepared at different deposition potentials. It can be seen that the deposition potential has a pronounced influence on the growth behavior of Cu_2−*x*_Se. At deposition potentials ranging from −0.07 V to −0.13 V, the film surfaces are predominantly composed of uniformly distributed nanosheet-like structures. This morphology can be attributed to the relatively slow deposition rate at less negative potentials, where the growth process is dominated by two-dimensional preferential crystal growth, leading to the formation of relatively flat sheet-like structures. As the deposition potential is further shifted to −0.16 V, spherical particles begin to emerge and gradually replace the original nanosheet morphology. When the deposition potential reaches −0.22 V and −0.28 V, the surfaces are mainly covered by spherical particles, indicating a transition of the Cu_2−*x*_Se growth mode from two-dimensional nanosheet growth to three-dimensional particle growth. This transition is likely associated with the significantly accelerated deposition rate at more negative potentials, which promotes rapid nucleation and particle accumulation, thereby favoring the formation of granular structures. These results demonstrate that the deposition potential can effectively regulate the microstructure of Cu_2−*x*_Se, which is closely related to the variation in nucleation and growth kinetics under different deposition conditions.

**Figure 4:**
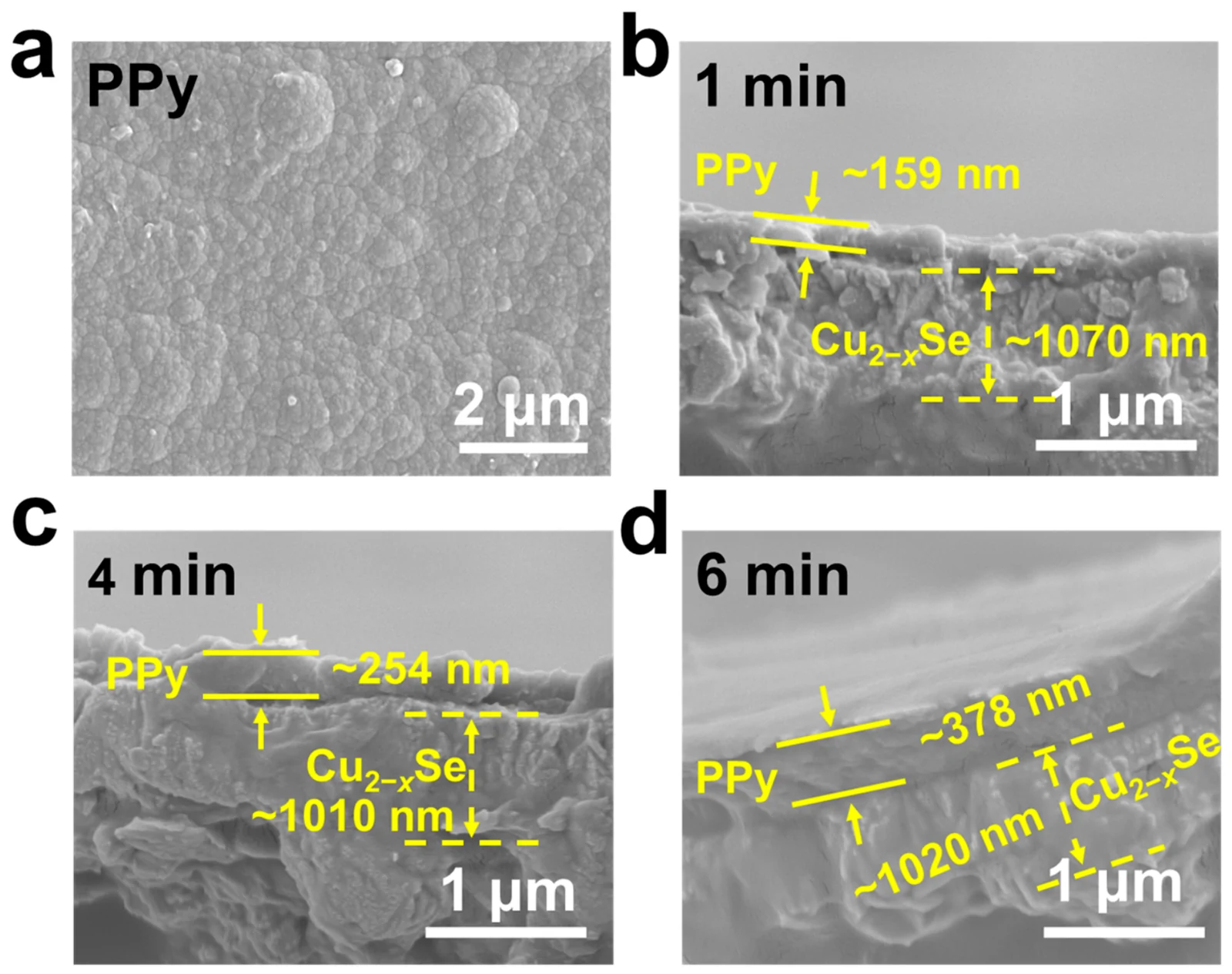
(**a**) SEM image of the pristine PPy film. Cross-sectional SEM images of PPy/Cu_2−*x*_Se composite films prepared with pyrrole polymerization times of (**b**) 1 min, (**c**) 4 min, and (**d**) 6 min.

The substrate structure plays a crucial role in governing the nucleation and growth behavior of inorganic thermoelectric materials. Figure S2 shows the surface morphologies of PPy/Cu_2−*x*_Se composite films prepared with different pyrrole polymerization times. All samples exhibit similar particle-like surface morphologies with no obvious differences, indicating that Cu_2−*x*_Se can be stably deposited on PPy layers with different thicknesses while maintaining a comparable growth mode. These results further demonstrate the good reproducibility and stability of the electrochemical synthesis process. Figure 4a shows the surface morphology of the PPy film together with the cross-sectional SEM images of PPy/Cu_2−*x*_Se composite films prepared with different polymerization times. As shown in Figure 4a, the electropolymerized PPy film exhibits a typical cauliflower-like morphology, forming a continuous and compact conductive layer [45]. To investigate the effect of polymerization time on the composite structure, cross-sectional observations were carried out, as shown in Figure 4b–d. The cross-sectional SEM image reveals a distinct bilayer structure consisting of a PPy layer and a Cu_2−*x*_Se layer, confirming the formation of an integrated organic/inorganic composite architecture. As the polymerization time increased from 1 min to 4 min and further to 6 min, the thickness of the PPy layer gradually increased from 159 ± 32 nm to 254 ± 45 nm and 378 ± 67 nm, respectively, whereas the thickness of the Cu_2−*x*_Se layer remained nearly constant at approximately 1.0 ± 0.1 μm with only minor variations. These results indicate that extending the Py polymerization time mainly promotes the growth of the PPy layer while exerting little influence on the subsequent deposition and thickness of the Cu_2−*x*_Se layer.

To further investigate the elemental distribution in the composite films, EDS elemental mapping was performed on the PPy/Cu_2−*x*_Se composite films, as shown in Figure S3. Figure S3a and Figure S3b present the EDS mapping results of the film surface and cross-section, respectively. Figure S3a shows the plane-view EDS elemental mapping of the PPy/Cu_2−*x*_Se composite film, where C, N, Cu, and Se are uniformly distributed throughout the sample region. Figure S3b presents the cross-sectional EDS mapping, in which the C and N signals originate from the PPy layer, while the Cu and Se signals correspond to the Cu_2−*x*_Se inorganic phase, indicating that Cu_2−*x*_Se was uniformly deposited on the PPy substrate and formed a stable composite structure. Figure S3c and Figure S3d show the quantitative EDS analysis results for the surface and cross-section, respectively. The surface EDS analysis reveals Cu and Se atomic contents of 66.50% and 33.50%, while the cross-sectional EDS analysis gives values of 66.77% and 33.23%, respectively. In both cases, the Cu/Se atomic ratio is close to 2:1, suggesting that the deposited copper selenide possesses a Cu-rich composition close to the Cu_2_Se-based copper selenide system. However, considering the inherent limitations of EDS analysis, such as its local detection nature, interaction volume effect, and quantitative uncertainty, the Cu/Se ratio alone cannot conclusively determine the exact stoichiometric composition or exclude the presence of intrinsic Cu vacancies commonly observed in copper selenide materials. Therefore, the composition and phase characteristics of the deposited copper selenide were further investigated by HRTEM, XRD, and XPS analyses.

**Figure 5:**
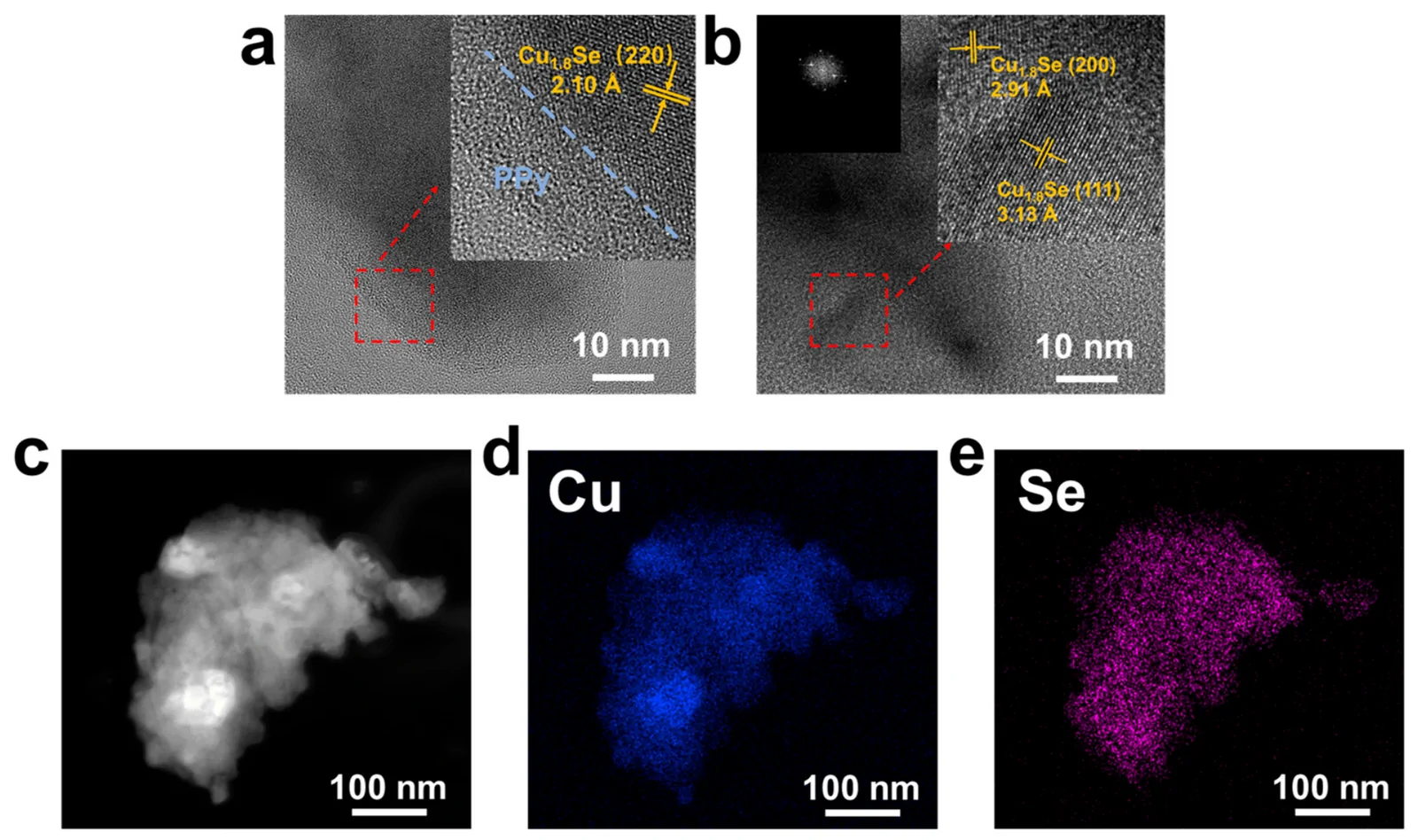
(**a**,**b**) HRTEM images of the PPy/Cu_2−*x*_Se composite film. (**c**–**e**) TEM-EDS elemental mappings of the PPy/Cu_2−*x*_Se composite film.

The interfacial structure and crystallographic features of the PPy/Cu_2−*x*_Se composite films were characterized by TEM analysis. As shown in Figure 5a, a distinct interface can be clearly observed between the PPy and Cu_2−*x*_Se regions. The brighter region corresponds to the PPy layer, while the darker region is attributed to the Cu_2−*x*_Se crystalline phase, indicating the successful deposition of Cu_2−*x*_Se on the PPy surface and the formation of an intimate composite structure. By comparing the obtained lattice spacings with the reported room-temperature reference cards, the observed lattice fringes show better agreement with the Cu_1.8_Se reference data, suggesting the formation of a Cu-deficient Cu_2−*x*_Se phase. Well-defined lattice fringes can also be observed in Figure 5a, with an interplanar spacing of approximately 2.10 Å, corresponding to the (220) plane of Cu_1.8_Se, Figure 5b further presents a high-resolution TEM image of the Cu_2−*x*_Se region, where two sets of clear lattice fringes with interplanar spacings of approximately 2.91 Å and 3.13 Å can be identified, corresponding to the (200) and (111) planes of Cu_1.8_Se, respectively [46,47]. In addition, the selected-area electron diffraction (SAED) patterns shown in the inset exhibit distinct diffraction spots, confirming the crystalline nature of the copper selenide phase and further supporting the successful synthesis of Cu_2−*x*_Se in the composite films. Figure 5c–e show the EDS elemental mapping results of the composite films. Cu and Se are uniformly distributed throughout the particle region without obvious elemental segregation, indicating the homogeneous growth of Cu_2−*x*_Se on the PPy substrate. Moreover, the spatial distributions of Cu and Se exhibit a high degree of overlap, demonstrating the uniform elemental distribution of the copper selenide phase within the composite films.

### 3.4. Analysis of XRD, Raman, and XPS Spectra

**Figure 6:**
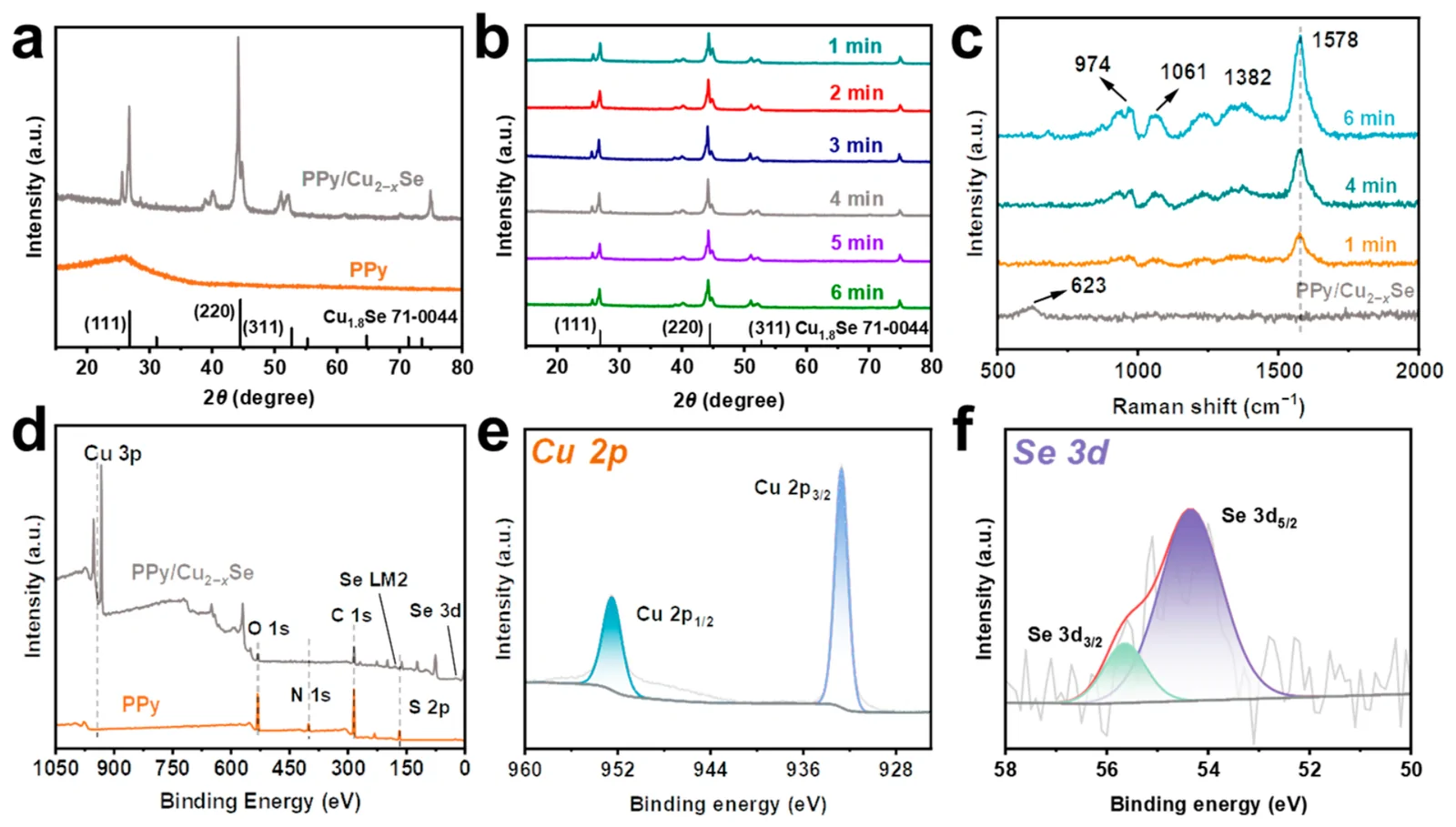
(**a**) XRD patterns of the pristine PPy film and the PPy/Cu_2−*x*_Se composite film. (**b**) XRD patterns of PPy/Cu_2−*x*_Se composite films prepared with different pyrrole polymerization times. (**c**) Raman spectra of PPy films and PPy/Cu_2−*x*_Se composite films prepared with different polymerization times. (**d**) XPS survey spectra of the pristine PPy film and the PPy/Cu_2−*x*_Se composite film. Deconvoluted XPS spectra of (**e**) Cu 2p and (**f**) Se 3d for the PPy/Cu_2−*x*_Se composite film.

To elucidate the effects of structural characteristics and interfacial chemical states on the thermoelectric performance of the PPy/Cu_2−*x*_Se composite films, XRD, Raman spectroscopy, and XPS analyses were conducted, as shown in Figure 6.

Figure 6a presents the XRD patterns of the pristine PPy film and the PPy/Cu_2−*x*_Se composite film. The pristine PPy film exhibits only a broad diffraction peak centered at approximately 25°, indicating its typical amorphous nature. After Cu_2−*x*_Se deposition, several distinct diffraction peaks corresponding to the (111), (220), and (311) planes emerge in the PPy/Cu_2−*x*_Se composite film; these diffraction peaks are in good agreement with the Cu_1.8_Se pattern (JCPDS No. 71-0044) [46,48]. Considering the intrinsic Cu vacancy characteristics of copper selenide compounds, the matching with the Cu_1.8_Se reference pattern indicates the formation of a Cu-deficient copper selenide structure, while the exact value of *x* cannot be determined solely from XRD analysis. Therefore, the deposited phase is described as Cu_2−*x*_Se in this work. To investigate the influence of pyrrole polymerization time on the crystal structure of Cu_2−*x*_Se, XRD measurements were further performed on samples prepared with different polymerization times, as shown in Figure 6b. All samples exhibit nearly identical diffraction peak positions and similar diffraction features, and no detectable secondary phases are observed. These results indicate that varying the polymerization time primarily affects the thickness of the PPy layer, while exerting negligible influence on the crystal structure and phase composition of the subsequently deposited Cu_2−*x*_Se layer.

Raman spectroscopy was conducted to probe the structural evolution of the samples, and the results are presented in Figure 6c. The pristine PPy films prepared with different polymerization times exhibit similar Raman spectra, indicating that variations in polymerization time do not alter the chemical structure of PPy. The characteristic peaks located at approximately 1382, and 1578 cm^−1^ can be assigned to C−N stretching vibration, and conjugated backbone vibration of PPy, respectively. Although slight variations in peak intensity are observed with increasing polymerization time, the peak positions remain essentially unchanged, suggesting that the molecular structure of PPy is well preserved. In contrast, the characteristic Raman bands of PPy almost disappear after Cu_2−*x*_Se deposition, and only a weak peak is observed at approximately 623 cm^−1^. According to the literature, this peak is generally associated with Cu_2_O-related vibrational modes and is likely attributed to slight surface oxidation of Cu_2−*x*_Se upon exposure to air [49,50]. The disappearance of the PPy Raman signals suggests that the deposited Cu_2−*x*_Se layer effectively covers the PPy surface. Overall, the Raman results provide further evidence for the successful construction of the PPy/Cu_2−*x*_Se composite structure.

To gain insight into the surface chemical composition and bonding states of the PPy/Cu_2−*x*_Se composite films, XPS analysis was conducted, as shown in Figure 6d. The survey spectrum of the pristine PPy film mainly consists of C, N, and O signals, where C and N originate from the PPy backbone, while the O signal is mainly attributed to adsorbed oxygen species or slight surface oxidation. In contrast, distinct Cu and Se peaks are observed in the survey spectrum of the PPy/Cu_2−*x*_Se composite film in addition to the C, N, and O signals, confirming the successful deposition of Cu_2−*x*_Se onto the PPy surface.

Figure 6e shows the high-resolution Cu 2p XPS spectrum of the PPy/Cu_2−*x*_Se composite film. Two characteristic peaks located at approximately 932.7 eV and 952.5 eV can be assigned to Cu 2p_3/2_ and Cu 2p_1/2_, respectively. The spin–orbit splitting energy between these two peaks is about 19.8 eV, which is in good agreement with the reported binding energies of Cu^+^ species, indicating that copper mainly exists in the Cu^+^ oxidation state [51]. Figure 6f presents the high-resolution Se 3d XPS spectrum of the PPy/Cu_2−*x*_Se composite film. After peak deconvolution, two characteristic peaks located at approximately 54.9 eV and 55.6 eV are observed, corresponding to the Se 3d_5/2_ and Se 3d_3/2_ spin–orbit components, respectively [52]. Both peaks can be attributed to the Se^2−^ species in the copper selenide lattice. Combined with the Cu^+^ species identified from the Cu 2p spectrum, these results confirm the formation of stable Cu–Se bonding during the electrodeposition process, supporting the formation of a copper selenide phase. This result further confirms the successful construction of the PPy/Cu_2−*x*_Se composite films.

### 3.5. Thermoelectric Properties

**Figure 7:**
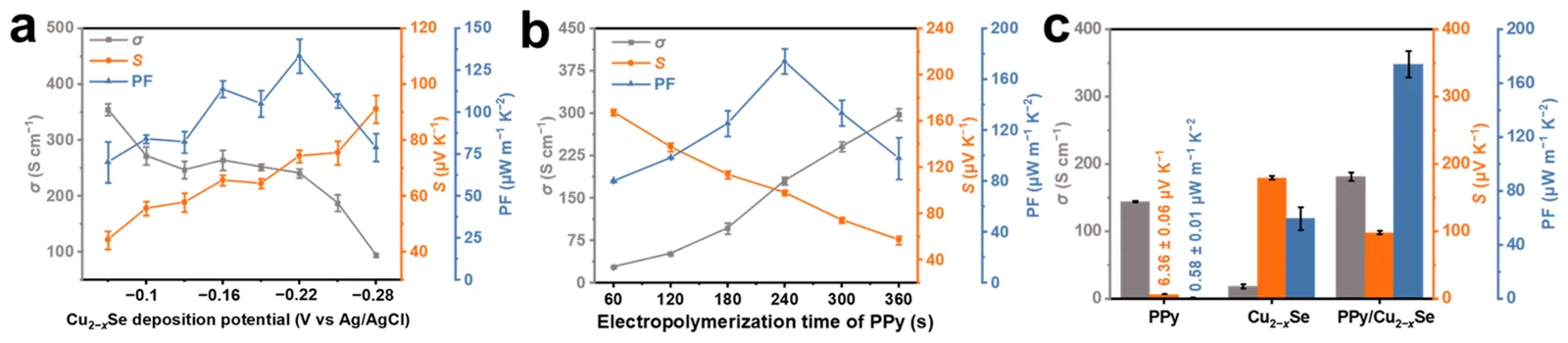
(**a**) Thermoelectric performance of PPy/Cu_2−*x*_Se composite films prepared at different deposition potentials. (**b**) Thermoelectric performance of PPy/Cu_2−*x*_Se composite films prepared with different PPy electropolymerization times. (**c**) Comparison of the thermoelectric performance between pristine PPy, Cu_2−*x*_Se, and PPy/Cu_2−*x*_Se composite films.

Figure 7a presents the influence of Cu_2−*x*_Se deposition potential on the thermoelectric performance of the PPy/Cu_2−*x*_Se composite films. As the deposition potential gradually shifts from −0.07 V to −0.28 V, the *σ* of the composite films continuously decreases from 354.78 ± 10.82 S cm^−1^ to 94.95 ± 3.17 S cm^−1^. In contrast, the *S* increases from 44.36 ± 3.18 μV K^−1^ to 91.18 ± 4.99 μV K^−1^. The evident trade-off between *σ* and *S* indicates that the deposition potential significantly affects the thermoelectric properties of the composite films. As a result, the PF first increases and then decreases as the deposition potential becomes more negative, reaching a maximum value of 133.55 ± 10.14 μW m^−1^ K^−2^ at −0.22 V. Although S further increases at more negative potentials, the pronounced decline in *σ* ultimately leads to PF deterioration. Therefore, −0.22 V is selected as the optimal deposition potential for subsequent Cu_2−*x*_Se deposition.

Figure 7b shows the influence of Py electropolymerization time on the thermoelectric performance of the PPy/Cu_2−*x*_Se composite films. As the polymerization time increases from 60 s to 360 s, the *σ* gradually increases from 28.48 ± 1.07 S cm^−1^ to 297.85 ± 10.55 S cm^−1^, while the S decreases from 167.7 ± 2.59 μV K^−1^ to 57.20 ± 3.72 μV K^−1^. These results indicate that the polymerization time mainly regulates the thickness of the PPy layer and the interfacial transport characteristics between PPy and Cu_2−*x*_Se. At short polymerization times, the incomplete PPy coverage and limited interfacial contact may hinder efficient charge transport across the PPy/Cu_2−*x*_Se interface, resulting in relatively low *σ*. With increasing polymerization time, a more continuous PPy layer is gradually formed, improving the interfacial coupling and providing more effective charge transport pathways, thereby increasing *σ*. Meanwhile, the increased interaction between PPy and Cu_2−*x*_Se interfaces can influence carrier scattering and transport behavior, leading to a decrease in *S*. When excessive PPy growth occurs, the thicker organic layer may introduce additional interfacial resistance and disturb the balance between *σ* and *S* [33]. Consequently, the PF first increases and then decreases as the polymerization time increases, reaching a maximum value of 174.05 ± 9.87 μW m^−1^ K^−2^ at 240 s. Therefore, 240 s is selected as the optimal electropolymerization time for Py.

Figure 7c compares the thermoelectric performance of pristine PPy, Cu_2−*x*_Se, and the PPy/Cu_2−*x*_Se composite film prepared under the optimal conditions (−0.22 V and 240 s). The pristine PPy film exhibits a low PF of only 0.58 ± 0.01 μW m^−1^ K^−2^, while the Cu_2−*x*_Se film exhibits a PF of 59.36 ± 8.39 μW m^−1^ K^−2^, with a *σ* of 18.52 ± 3.18 S cm^−1^ and an *S* of 179.31 ± 2.67 μV K^−1^. The PPy/Cu_2−*x*_Se composite film achieves a PF of 174.05 ± 9.87 μW m^−1^ K^−2^, corresponding to an enhancement of nearly 300-fold compared with pristine PPy. These results demonstrate that the deposition of the Cu_2−*x*_Se layer constructs a unique PPy/Cu_2−*x*_Se composite architecture, which enhances the overall thermoelectric performance through the combined contributions of both components, while the comparison with pristine PPy and Cu_2−*x*_Se films further confirms that the PPy/Cu_2−*x*_Se bilayer architecture enables a more balanced optimization of *σ* and *S*. The improved thermoelectric performance originates from the complementary contributions of PPy and Cu_2−*x*_Se, where PPy provides efficient electrical transport and mechanical flexibility, while Cu_2−*x*_Se contributes to the enhanced *S*.

The enhanced thermoelectric performance can be further understood from the perspective of carrier transport behavior. Compared with pristine PPy, the introduction of the Cu_2−*x*_Se layer significantly enhances the *S* of the composite film, while the PPy layer maintains efficient electrical transport due to its relatively high *σ*. This complementary contribution of the two components enables the PPy/Cu_2−*x*_Se composite film to achieve a more favorable balance between *σ* and *S*. According to thermoelectric transport theory, *σ* is governed by both carrier concentration and carrier mobility, whereas the *S* is closely related to the carrier transport state [53]. Therefore, the enhanced PF of the PPy/Cu_2−*x*_Se composite film can be attributed to the optimized carrier transport behavior induced by the PPy/Cu_2−*x*_Se interface, where carrier concentration, mobility, and carrier scattering processes are collectively regulated. In addition, the *κ*_⊥_ of the optimized PPy/Cu_2−*x*_Se composite film was measured to be 0.30 W m^−1^ K^−1^. Due to the considerable difficulty in accurately determining the in-plane thermal conductivity (*κ*_//_) of thin flexible films, only *κ*_⊥_ was measured in the present work. Based on the measured thermoelectric parameters, the maximum estimated *ZT* value at room temperature was calculated to be 0.17. This value is provided for reference only, since the anisotropic structure of organic/inorganic composite films generally results in *κ*_⊥_ being lower than *κ*_//_.

### 3.6. Thermal Stability, Flexibility, Environmental Stability and Performance of TEG

**Figure 8:**
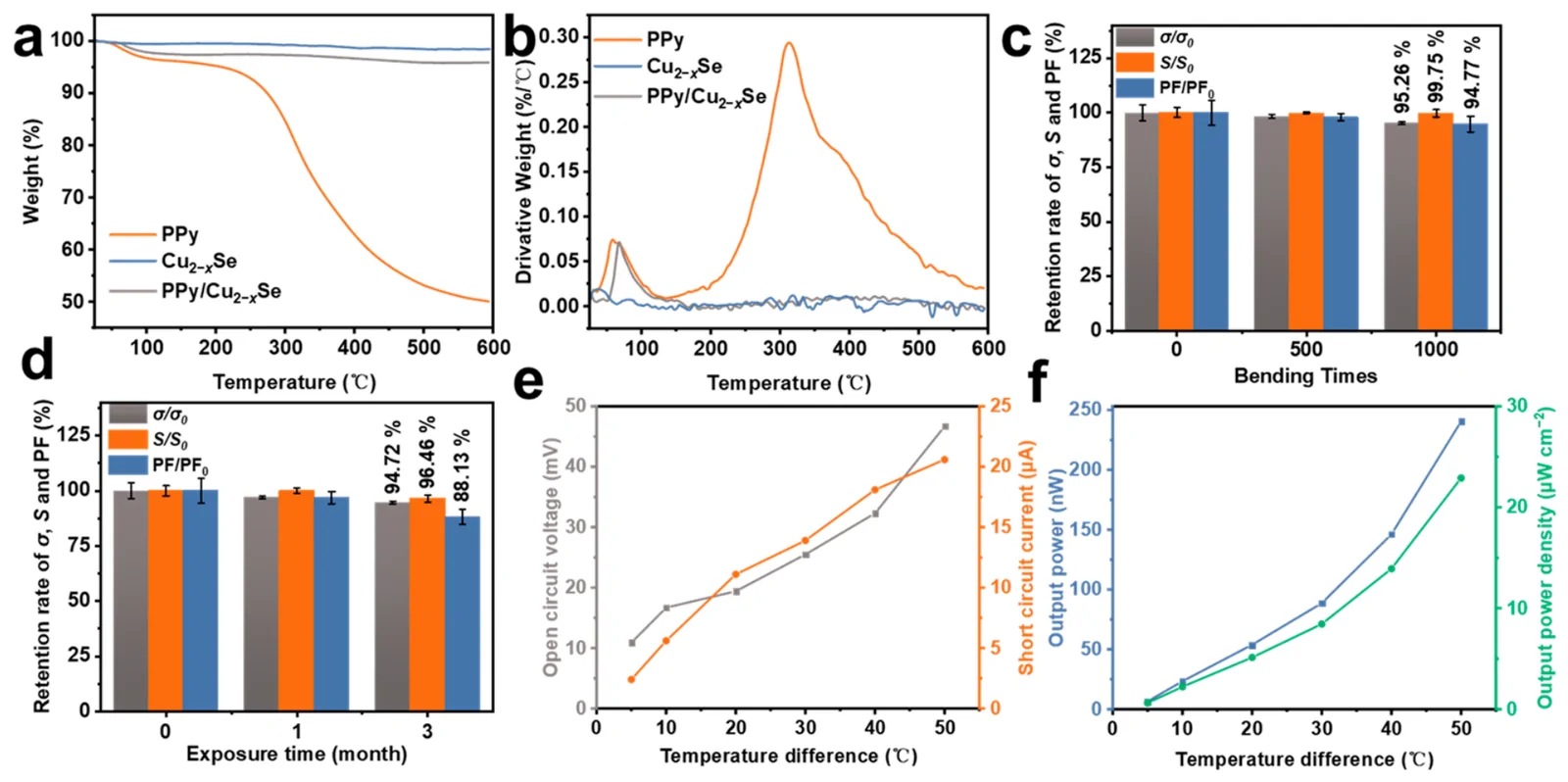
(**a**) TG curves and (**b**) DTG curves of pristine PPy, Cu_2−*x*_Se and PPy/Cu_2−*x*_Se composite films. (**c**) Bending cycle test and (**d**) environmental stability test of the PPy/Cu_2−*x*_Se composite films. (**e**) *V*_oc_ and *I*_sc_ of the flexible TEG under different temperature gradients. (**f**) Output power and output power density of the flexible TEG under different temperature gradients.

To evaluate the thermal stability of the composite films, thermogravimetric analysis (TGA) and derivative thermogravimetry (DTG) were performed on pristine PPy, pristine Cu_2−*x*_Se, and PPy/Cu_2−*x*_Se composite samples, and the results are shown in Figure 8a,b. The pristine PPy film exhibits significant weight loss throughout the heating process. The weight loss in the low-temperature region is mainly attributed to the evaporation of adsorbed water and residual low-molecular-weight species, while a rapid weight loss is observed in the temperature range of approximately 250−450 °C, corresponding to the thermal decomposition of the PPy molecular chains. A pronounced decomposition peak is observed in the DTG curve, further confirming the degradation of the PPy backbone within this temperature range. In contrast, pristine Cu_2−*x*_Se exhibits only slight weight loss within the investigated temperature range, which may be associated with the thermal decomposition of Cu_2−*x*_Se accompanied by Se sublimation. Meanwhile, no obvious decomposition peak is observed in the DTG curve, indicating that no severe thermal degradation occurs under the present TGA conditions. The PPy/Cu_2−*x*_Se composite film exhibits improved thermal stability compared with pristine PPy, with the characteristic PPy degradation peak between 250 and 450 °C significantly suppressed. Although the high thermal stability and large mass fraction of Cu_2−*x*_Se contribute to the reduced weight loss of the composite film, the disappearance of the PPy-associated degradation peak suggests that the improved thermal stability is not solely caused by a simple dilution effect. These results demonstrate that the incorporation of Cu_2−*x*_Se effectively modifies the thermal degradation behavior of the PPy/Cu_2−*x*_Se composite architecture.

For flexible thermoelectric devices, excellent mechanical flexibility and environmental stability are essential prerequisites for practical applications in wearable electronic systems. Figure 8c and Figure 8d show the performance evolution of the PPy/Cu_2−*x*_Se composite film during bending cycle and environmental stability tests, respectively. The bending test was conducted at a fixed bending radius of 5 mm. After 1000 bending cycles, the *σ*, *S*, and PF retained 95.26%, 99.75%, and 94.77% of their initial values, respectively. In addition, after exposure to ambient conditions for three months, the PPy/Cu_2−*x*_Se composite film still retained 94.72% of its initial *σ*, 96.46% of its initial *S*, and 88.13% of its initial PF, demonstrating relatively good environmental stability. These results indicate that the composite film possesses excellent flexibility and environmental stability, highlighting its promising potential for applications in flexible wearable thermoelectric devices.

A flexible TEG was assembled using the optimized PPy/Cu_2−*x*_Se composite film as the p-type component and polyethyleneimine (PEI)-treated SWCNTs as the n-type component (as shown in Figure S4). Its output performance under different temperature gradients was evaluated, and the results are presented in Figure 8e,f. As the temperature difference (Δ*T*) increased, both the *V*_oc_ and *I*_sc_ exhibited a steady increasing trend, as shown in Figure 8e. At the maximum tested Δ*T*, the device delivered a *V*_oc_ of 46.7 mV and an *I*_sc_ of 20.6 μA, indicating that the composite film can effectively convert a temperature gradient into electrical energy. Figure 8f presents the variation in output power and power density with temperature difference. With increasing ΔT, the output power continuously increased and reached approximately 240.5 nW at the maximum tested temperature gradient, corresponding to a power density of 22.91 μW cm^−2^. These results demonstrate the excellent thermoelectric energy conversion capability of the electrochemically fabricated PPy/Cu_2−*x*_Se composite film. Despite the relatively simple device architecture of the TEG constructed in this work, stable voltage and power outputs were achieved, validating the promising potential of this composite film for low-grade waste heat harvesting and self-powered wearable electronic applications.

## 4. Conclusions

In summary, PPy/Cu_2−*x*_Se composite thermoelectric films were successfully fabricated through a combination of electrochemical polymerization and electrodeposition, enabling effective interfacial coupling between conducting polymers and inorganic thermoelectric materials. By tuning the deposition potential of Cu_2−*x*_Se and the polymerization time of pyrrole, the microstructure, composite architecture, and thermoelectric transport properties of the films were effectively regulated. The optimized PPy/Cu_2−*x*_Se composite film, prepared at a deposition potential of −0.22 V and a polymerization time of 240 s, achieved a maximum PF of 174.05 μW m^−1^ K^−2^, which is nearly 300 times higher than that of pristine PPy films. Meanwhile, the composite film exhibited a low *κ* of 0.30 W m^−1^ K^−1^ and an estimated room-temperature *ZT* value of 0.17, demonstrating the coexistence of favorable electrical transport and suppressed thermal transport. In addition, the composite films showed excellent thermal stability, mechanical flexibility, and environmental stability. The flexible thermoelectric device assembled from the optimized PPy/Cu_2−*x*_Se composite films delivered an open-circuit voltage of 46.7 mV, a short-circuit current of 20.6 μA, and a maximum output power of 240.5 nW under a temperature difference of 50 K, corresponding to a power density of 22.91 μW cm^−2^. This work provides a new strategy for the rational design and controllable electrochemical fabrication of flexible organic/inorganic thermoelectric composites.

## Data Availability

The original contributions presented in this study are included in the article/Supplementary Materials. Further inquiries can be directed to the corresponding authors.

## Supplementary Materials

The following supporting information can be downloaded at: https://www.mdpi.com/article/10.3390/ma19163496/s1, Figure S1: Schematic of the Seebeck coefficient and electrical conductivity measurement system; Figure S2: SEM images of PPy/Cu_2−*x*_Se composite films at different Py polymerization times; Figure S3: Plane-view (a,c) and cross-sectional (b,d) EDS elemental mapping images and corresponding quantitative EDS spectra of the PPy/Cu_2−*x*_Se composite film; Figure S4: Schematic diagram of prototype TEG; Table S1: Comparison of thermoelectric performance and device characteristics of PPy/Cu_2−*x*_Se film with previously reported flexible thermoelectric films. References [31,32,54,55,56,57,58,59] are cited in the Supplementary Materials.
