# Supplementary material for: Electrochemical Synthesis of Polypyrrole/Cu2−xSe Composites for Enhanced Thermoelectric Performance
Source: Materials (Basel). 2026 Aug 18;19(16):3496. doi: 10.3390/ma19163496 (PMC13514461; doi:10.3390/ma19163496)
Supplement: Supplementary file 1 [file materials-19-03496-s001.zip › materials-4446383-supplementary.pdf]

Supporting Information

Electrochemical Synthesis of Polypyrrole/$Cu_{2-x}Se$ Composites for Enhanced Thermoelectric Performance

Yunfei Cai [1], Caiyan Gao [2,*] and Cun-Yue Guo [1,*]

1 School of Chemical Sciences, University of Chinese Academy of Sciences, Beijing 101408, P. R. China
2 Beijing National Laboratory for Molecular Sciences (BNLMS), Key Laboratory of Green Printing, Institute of Chemistry, Chinese Academy of Sciences, Beijing 100190, P. R. China
* Correspondence: gaocaiyan@iccas.ac.cn (C.G.); cyguo@ucas.ac.cn (C.-Y.G.)

Experimental

1.1. Materials

Unless otherwise stated, all commercially available reagents and solvents were used directly without further purification. Pyrrole (Py, 99%) was purchased from Shanghai Aladdin Biochemical Technology Co., Ltd. Selenium oxide (SeO_2, 99%) and p-toluenesulfonic acid (p-TSA, 98%) were purchased from Beijing Innochem Science & Technology Co., Ltd. Copper sulfate pentahydrate ($CuSO_4 \cdot 5H_2O$, 99%) was purchased from Shanghai Macklin Biochemical Co., Ltd.

1.2. Characterization

The electrosynthesis and linear sweep voltammetry (LSV) measurements were conducted using an electrochemical workstation (CHI660E, Chenhua Instruments Co., Shanghai, China). Stainless steel (SS) was used as the working electrode for the electrochemical polymerization of pyrrole, followed by the electrodeposition of $Cu_{2-x}Se$. Raman spectra were obtained using a Labram Odyssey Raman system (HORIBA Ltd., Kyoto, Japan) at an excitation wavelength of 532 nm. X-ray photoelectron spectroscopy (XPS) measurements were conducted with an ESCALAB QXi X-ray photoelectron spectrometer (Thermo Fisher Scientific Ltd., Waltham, MA, USA). X-ray diffraction (XRD) was conducted using a D8-ADVANCE diffractometer (Bruker, Ltd., Karlsruhe, Germany). Scanning electron microscopy (SEM) and energy-dispersive spectroscopy (EDS) analyses were performed using an SU8010 scanning electron microscope (HITACHI Ltd., Tokyo, Japan). High-resolution transmission electron microscopy (HRTEM) was performed using a field emission transmission electron microscope (HT-7700Exalens, Tokyo, Japan). TG measurements were carried out on a thermo-gravimetric analysis analyzer (TGA, STA200, HITACHI Ltd., Tokyo, Japan) over a temperature range from room temperature to 600 ℃ at a heating rate of 10 °C min^{-1}. The thermal stability of the films was evaluated by TGA/DTA using approximately 5–10 mg of sample in an alumina crucible under a nitrogen atmosphere with a gas flow rate of 100 mL min^{-1}.

The thermoelectric parameters of the films were tested at room temperature using MRS-3RT thermoelectric parameter test system (JouleYacht Ltd., Wuhan, Hubei, China). The detailed measurement procedure is illustrated in Figure S1. The thickness of the composite thin film was measured by SEM. The error bars were calculated based on the standard deviation of at least three independent measurements to ensure statistical reliability. The out-of-plane thermal conductivity ($\kappa_{\perp}$) was measured by a transient hot wire method using a thermal conductivity tester (TC3000E, Xiatech Electronics Co., Ltd., Hangzhou,

Zhejiang, China). The open-circuit voltage (V_{oc}), short-circuit current (I_{sc}), and output power of the thermoelectric generator (TEG) were tested at different temperature gradients using a VC890C multimeter (Yisheng Victory Technology Co. Ltd., Shenzhen, Guangdong, China). Unless otherwise specified, all structural and chemical characterizations, including SEM, TEM, XRD, Raman, XPS, and TGA/DTG measurements, were performed using the as-deposited films directly grown on SS substrates. The films used for thermoelectric performance measurements and flexible TEG assembly/testing were transferred onto adhesive tape to enable flexible device fabrication and evaluation.

1.3. Electrochemical synthesis of PPy films

PPy films were synthesized in a conventional three-electrode system using a stainless-steel (SS) plate (4×4 cm^2) as the working electrode (WE), an Ag/AgCl electrode as the reference electrode (RE), and a platinum sheet (99.99%, 2×2 cm^2) as the counter electrode (CE). The electrolyte consisted of an aqueous solution containing 0.5 M *p*-toluenesulfonic acid (*p*-TSA) and 0.2 M pyrrole (Py). Electropolymerization of PPy was carried out under a constant current of 3.2 mA (corresponding to a current density of 0.8 mA cm^{-2}) for different polymerization times of 1, 2, 3, 4, 5, and 6 min. After electropolymerization, the obtained PPy films were thoroughly rinsed with ethanol and deionized water to remove residual monomers and adsorbed electrolytes, followed by vacuum drying at 60 ℃ prior to further use.

1.4. Electrochemical synthesis of PPy/$Cu_{2-x}Se$ composite films

PPy/$Cu_{2-x}Se$ composite films were prepared by potentiostatic electrodeposition in a conventional three-electrode cell. The PPy-coated stainless-steel substrate prepared as described above was used as the WE. The electrolyte consisted of an aqueous solution containing 8 mM copper sulfate pentahydrate ($CuSO_4 \cdot 5H_2O$), 3 mM selenium dioxide (SeO_2), and 0.05 M *p*-toluenesulfonic acid (*p*-TSA). Cu_2Se was electrodeposited at room temperature under applied potentials of −0.07, −0.10, −0.13, −0.16, −0.19, −0.22, −0.25, and −0.28 V for a fixed deposition time of 1.5 h. After deposition, the thickness of the Cu_2Se layer was approximately 1 μm. The resulting samples were thoroughly rinsed with ethanol and deionized water to remove residual electrolyte, followed by vacuum drying at 60 ℃ for 12 h. Finally, the PPy/$Cu_{2-x}Se$ composite films were peeled from the stainless-steel substrates using adhesive tape to obtain free-standing films for subsequent thermoelectric measurements [1].

For comparison, the pristine PPy film was also prepared by depositing PPy for 30 min in a three-electrode cell system in the same manner as described above, except that an indium tin oxide (ITO) was used as the WE instead of the stainless-steel plate.

1.5. Preparation of n−Type SWCNTs film [2]

5 mg SWCNTs and 100 mg PEI were dispersed in 25 mL of methanol. The resulting suspension was sonicated using an ultrasonic homogenizer (JY96-11N, Xinzi Biotechnology Co., Ltd., China) in an ice-water bath at 65 W for 60 min to prevent overheating. After sonication, the PEI/SWCNT composite films were obtained by vacuum filtration. The films were frozen with liquid nitrogen and subsequently dried in a vacuum freeze dryer at −60 ℃ for 24 h to yield the dried n-type SWCNT composite films.

1.6. Preparation of thermoelectric generator (TEG)

The TEG was assembled using p-type PPy/$Cu_{2-x}Se$ composite films and n-type PEI/SWCNT films. The p-type and n-type films were first cut into strips with dimensions of 5 mm × 15 mm, and copper sheets were cut into 5 mm-wide strips as electrical connectors. The film strips were then sequentially arranged in a p–n–p–n configuration and fixed

onto a polyimide (PI) substrate using double-sided adhesive tape. Seven pairs of p–n legs were electrically connected in series with conductive silver paste, while copper strips attached to the top and bottom ends of the device served as conductive pathways. Finally, the conductive silver paste was cured in a vacuum oven at 60 ℃ for 30 min, and the assembled device was encapsulated with transparent adhesive tape.

1.7. Thermoelectric performance test

The electrical conductivity and Seebeck coefficient of the composite films were simultaneously measured at room temperature using a commercial thermoelectric measurement system (MRS-3RT, Joule Yacht, China). For thermoelectric measurements, the prepared films were cut into strip-shaped samples with dimensions of 5 × 15 mm.

The Seebeck coefficient was measured using a dynamic method. During the measurement process, one side of the sample was periodically heated for a certain period of time to generate a series of gradually increasing temperature differences between the hot and cold ends of the sample (ΔT_1, ΔT_2, …, ΔT_n). The corresponding thermoelectric voltages (ΔV_1, ΔV_2, …, ΔV_n) were simultaneously recorded. The Seebeck coefficient was then determined from the slope (K) of the linear relationship between ΔV and ΔT by using least-squares fitting, minimum residual fitting, or the BiSquare fitting method.

The electrical conductivity was measured using a standard four-probe method, in which a constant current was applied through the two outer probes while the voltage was measured using the two inner probes. The resistivity (ρ) was calculated according to $\rho = \frac{VA}{IL}$, where V is the measured voltage, A is the cross-sectional area of the sample, I is the applied current, and L is the distance between the inner probes. The electrical conductivity (σ) was then obtained from $\sigma = \frac{1}{\rho}$.

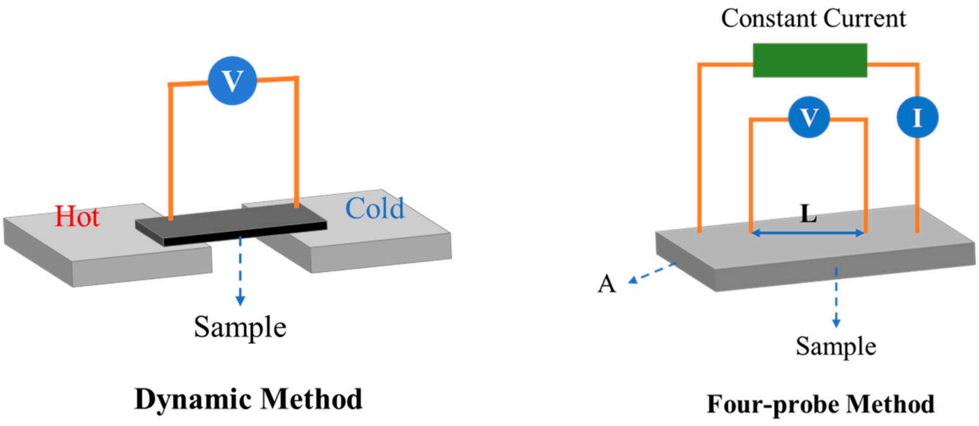

Figure S1. Schematic of the Seebeck coefficient and electrical conductivity measurement system.

Table S1. Comparison of thermoelectric performance and device characteristics of PPy/$Cu_{2-x}Se$ film with previously reported flexible thermoelectric films.

Materials [Ref]	PF ($\mu W\ m^{-1} K^{-2}$)	κ ($W\ m^{-1} K^{-1}$)	Output power (nW)
PPy/Te [3]	4.02	–	–
PEDOT/CNTs [4]	131.1	–	–
PPy/Ag [5]	0.62	–	–
PEDOT: PSS/SnSe [6]	36.20 ± 0.21	–	17.92
SWCNT/Cu_2Se [7]	26.0	–	62
PPy/Bi-Te/PPy [8]	244 ± 6	0.527 ± 0.001	172
PPy/$Cu_{2-x}Se$ [This work]	174.05 ± 9.87	0.30	240.5

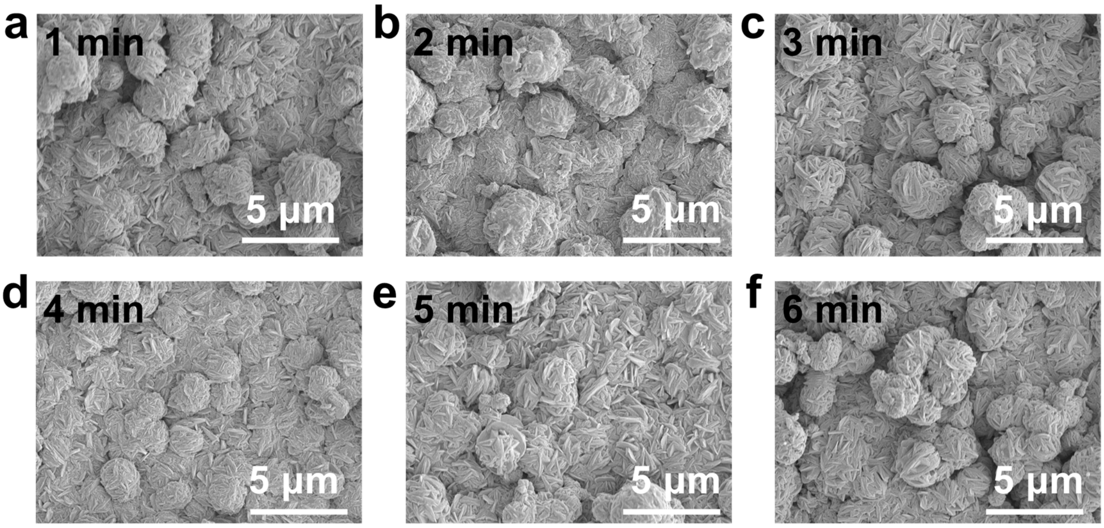

Figure S2. SEM images of PPy/$Cu_{2-x}Se$ composite films at different Py polymerization times.

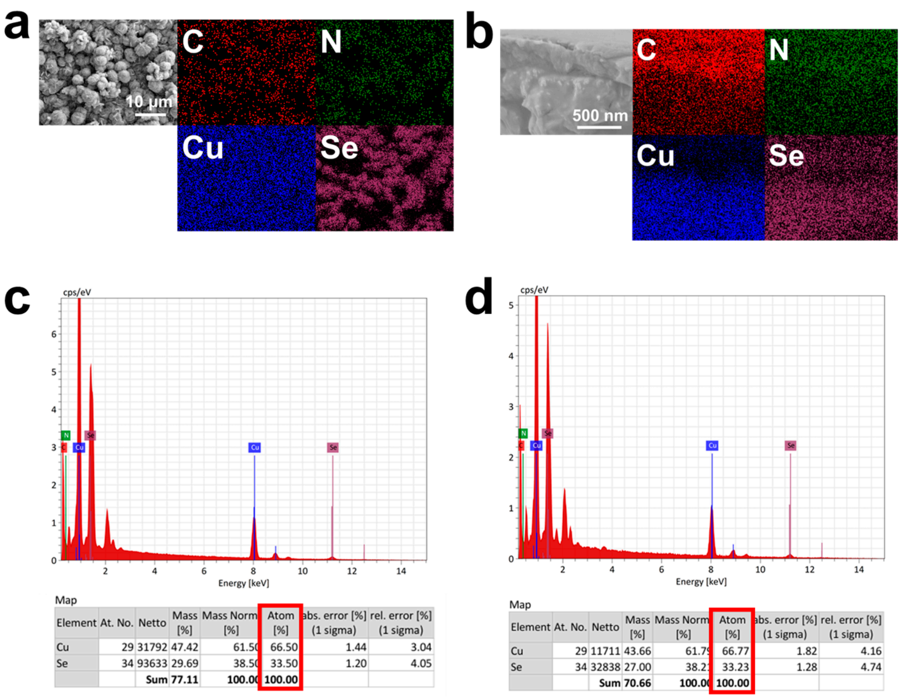

Figure S3. Plane-view (**a**,**c**) and cross-sectional (**b**,**d**) EDS elemental mapping images and corresponding quantitative EDS spectra of the PPy/$Cu_{2-x}Se$ composite film.

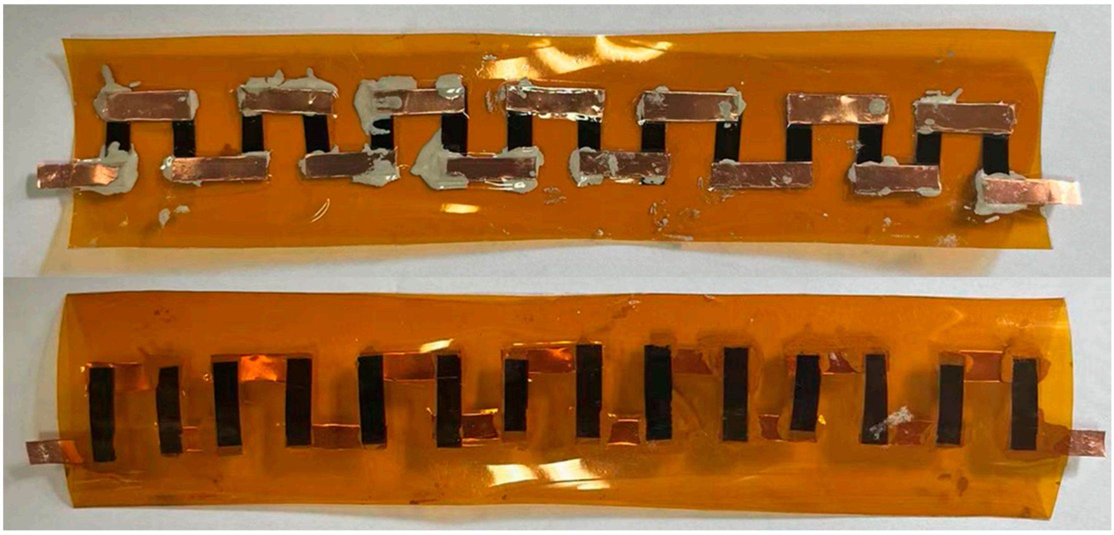

Figure S4. Schematic diagram of prototype TEG.

References

1. Li, Y.; Gao, C.-Y.; Fan, X.-H.; Yang, L.-M. Full-Electrochemical Construction of High-Performance Polypyrrole/Tellurium Thermoelectrical Nanocomposites. *ACS Appl. Mater. Interfaces* **2022**, *14*, 10815–10824. https://doi.org/10.1021/acsami.1c22731.
2. Wu, X.; Liu, P.; Huo, B.; Li, Y.; Liu, S.; Lv, Y.; Guo, C.-Y. High Power Factor of p- and n-Type Single-walled Carbon Nanotubes by Solution Processing. *Energy Mater. Adv.* **2024**, *5*, 0110. https://doi.org/10.34133/energymatadv.0110.
3. Liu, M.; Li, M.; Wu, R.; Liu, P.; Liu, C. Improved Thermoelectric Performance of Flexible Polypyrrole/Tellurium Flexible Films Prepared by Two-Step Electrochemical Deposition. *Int. J. Electrochem. Sci.* **2022**, *17*, 221291, https://doi.org/10.20964/2022.12.69.
4. Nasiri, M.A.; Tong, S.Y.; Cho, C.; Gómez, C.M.; Cantarero, A.; Culebras, M. Synthesis of PEDOT/CNTs Thermoelectric Thin Films with a High Power Factor. *Materials* **2024**, *17*, 1121. https://doi.org/10.3390/ma17051121.
5. Yin, M.; Du, H.; Liu, Y.; Li, L.; Yu, X. Improved Thermoelectric Performance of Flexible Film Based on Polypyrrole/Silver Nanocomposites. *J. Electron. Mater.* **2022**, *51*, 1061–1067. https://doi.org/10.1007/s11664-021-09356-y.
6. Ma, J.; Xu, Y.; Nan, Z.; Wang, Y.; Han, Y.; Zhao, B.; Zhao, M.; Wang, H. Modulation for Seebeck coefficient and power factor of flexible PEDOT:PSS films by incorporating SnSe nanosheets and solvents treatment. *J. Alloy. Compd.* **2025**, *1022*, 179793. https://doi.org/10.1016/j.jallcom.2025.179793.
7. Liu, Y.-M.; Shi, X.-L.; Li, M.; Cao, T.; Wu, H.; Wang, D.-Z.; Wu, T.; Yin, L.-C.; Liu, W.-D.; Chen, Z.-G.; et al. Cu_2Se hybridization advances high-performance flexible single-walled carbon nanotube based thermoelectrics. *J. Colloid Interface Sci.* **2025**, *698*, 138049. https://doi.org/10.1016/j.jcis.2025.138049.
8. Li, Y.; Chen, Z.-P.; Gao, C.-Y.; Li, H.-P.; Fan, X.-H.; Cao, X.; Yang, L.-M. Electrochemical assembly of flexible PPy/Bi–Te alloy/PPy thermoelectric composite films with a sandwich-type structure. *New J. Chem.* **2023**, *48*, 1634–1641, https://doi.org/10.1039/d3nj04785g.
